# Climate change research and the search for solutions: rethinking interdisciplinarity

**DOI:** 10.1007/s10584-021-03237-3

**Published:** 2021-10-18

**Authors:** E. Lisa F. Schipper, Navroz K. Dubash, Yacob Mulugetta

**Affiliations:** 1 Environmental Change Institute, University of Oxford, Oxford, OX1 3QY UK; 2 Centre for Policy Research, Dharma Marg, Chanakyapuri, New Delhi 110 021 India; 3 Department of Science, Technology, Engineering & Public Policy, University College London, Gower Street, London, WC1E 6BT UK

**Keywords:** Interdisciplinarity, Climate change research, Reductionism, Social science, Climate solutions

## Abstract

Growing political pressure to find solutions to climate change is leading to increasing calls for multiple disciplines, in particular those that are not traditionally part of climate change research, to contribute new knowledge systems that can offer deeper and broader insights to address the problem. Recognition of the complexity of climate change compels researchers to draw on interdisciplinary knowledge that marries natural sciences with social sciences and humanities. Yet most interdisciplinary approaches fail to adequately merge the framings of the disparate disciplines, resulting in reductionist messages that are largely devoid of context, and hence provide incomplete and misleading analysis for decision-making. For different knowledge systems to work better together toward climate solutions, we need to reframe the way questions are asked and research pursued, in order to inform action without slipping into reductionism. We suggest that interdisciplinarity needs to be rethought. This will require accepting a plurality of narratives, embracing multiple disciplinary perspectives, and shifting expectations of public messaging, and above all looking to integrate the appropriate disciplines that can help understand human systems in order to better mediate action.

## Urgency to solve climate change

Following the launch of the Intergovernmental Panel on Climate Change (IPCC) Special Report on 1.5C (IPCC 2018), pressure to take action on climate change has escalated significantly (Boykoff and Pearman 2019). While this attention is to be welcomed, it has implications for researchers of, and research on, climate change. A clear push is evident from within both the scientific and policy communities to get scientists to move beyond merely providing knowledge about climate change, to also helping society define the solutions (Haasnoot et al. 2020; Callaghan et al. 2020). Additionally, the solutions focus mirrors—and drives—civil society pressure for governments to take action on the climate crisis (Fisher and Nasrin 2020). This introduces new challenges for the ways in which science on climate change is undertaken and how the knowledge gained is used in the world of practice and policy (Hulme 2020). In particular, the search for solutions to climate change forces us to examine the way different disciplines interact in this process, most prominently through interdisciplinary research approaches (Castree et al. 2014).

In response to pressure for concrete, urgent, and actionable information, however, researchers often shear away detail, and pick one of a number of alternative messages to unite behind. The emerging narrative of ‘listen to the science’ unfortunately reinforces the message that discreet, clear, and neutral solutions readily exist (Evensen 2019). This is underpinned by a strong belief that science needs to be represented and communicated in easy-to-digest ‘bite-size’ statements targeting a policy audience, commonly believed to be working under time duress. The growing tendency for complex scientific findings to be summarized into single sentences for policy briefs is case-in-point. These communiqués are often simplified, tend to emphasize quantitative detail, and fail to do justice to the messiness of climate change impacts as they are experienced by people and ecosystems in different parts of the globe (Castán Broto 2020; Hulme 2011a). Because they also abstract from political context, they avoid grappling with the reality of implementation challenges, and reflecting essential ambiguities that would raise the level of debate and reflection.

The critique that climate change research needs to be open to ways of knowing other than the natural sciences is not new (Shah 2020; Heymann 2019; Rigg and Reyes Mason 2018; Victor 2015; Barnes et al. 2013; Hulme 2011a and b; Jasanoff 2010), nor are the calls for interdisciplinarity (Bhaskar et al. 2010; Simonovic and Davies 2006) and inter-epistemology (Murphy 2011). Interdisciplinarity is understood as the collective efforts to tackle a single issue from multiple disciplinary perspectives. In particular, this cuts across the natural sciences, the social sciences, and the humanities. Our purpose here is to take that message further. We argue that in the push for solutions to climate change, knowledge on climate change is expressed in simplified and narrow ways that privileges predictive natural sciences over interpretative qualitative social sciences and humanities, even when this knowledge is generated in ostensibly interdisciplinary interactions.

Yet, because solutions are context-dependent and therefore not universal, the interpretive sciences are necessary to identify feasible and effective solutions. For example, a qualitative and locally informed assessment of vulnerability in a specific location can help avoid the maladaptation that otherwise risks resulting from poorly designed blue-print adaptation strategies (Eriksen et al. 2021). Consequently, we need new ways of generating and communicating knowledge on climate change through an interdisciplinarity that does not limit our visions nor narrow the evidence base needed for problems rooted in the deep relationship between the biosphere and human systems. We underscore the intricacy between interdisciplinarity and transdisciplinarity influenced by the need for policy relevance that is based on the assessment of scientific knowledge, which creates a unique context for reflection on which knowledge is needed to address the climate crisis. This commentary thus offers a cautionary tale about the consequences of conducting nominally interdisciplinary climate change research and assessment under the pressure to generate solutions. We explain why pressure for action leads to reductionist approaches, comment critically on interdisciplinarity as it is currently practised, and offer reflections on a way forward for rethinking interdisciplinarity.

## Pressure for solutions leads to reductionism

Embracing the complexity of climate change research means reflecting both the natural and the social world, investigating the ways we give meaning to each and examining how they interact with each other. This requires conversations across disciplines to allow for answering questions that a single disciplinary lens cannot fully explain. It also means dealing with the inherent epistemic tensions between the disciplines and greater engagement with difference, which can be a source of creativity and deep learning. There is now increasing agreement that qualitative approaches and diverse ontological models are also needed to understand climate change in specific contexts (Nightingale et al. 2020; Goldman et al. 2018; Hulme 2010). Such perspectives bring out a more complete picture of climate change impacts, the drivers of vulnerability, and opportunities for adaptation and mitigation (Howarth et al. 2018). But this can lead to messy, contradictory information that is at odds with the type of evidence that decision-makers require for their applied world. For example, rather than a narrative about who is more vulnerable to climate change in a specific location, decision-makers ask for vulnerability indicators that lump diverse populations together in typologies, forcing categorization even when some individuals would span multiple categories. As a consequence, there remains a strong tendency to focus on reductionist messages, i.e. ‘clean’ narratives that offer discreet pathways (Rigg and Reyes Mason 2018), by definition excluding alternative, conflicting approaches that stem from diverse knowledges, including indigenous knowledge (Murphy 2011; Ford et al. 2016; Farbotko and Lazrus 2012). To illustrate these points, we examine two ways in which current representations of climate change knowledge sideline interpretivist disciplines: the way that numbers embody the process of reductionism, and how lack of epistemological diversity also reinforces reductionism and prevents interdisciplinarity from taking place.

### Numbers—not stories

Visual representations of science have a long history (Trumbo 2000). Numbers and graphics, which underpin such representations, undoubtedly have an impact in policy conversations, particularly by concretizing complicated information for non-experts and are frequently seen as a way to convey both quantitative and qualitative knowledge on climate change. The result is that often numbers get prioritized over stories. Indeed, the desire by governments for IPCC figures to express ideas simply (Thoni and Livingston 2019) suggests that decision-makers are attuned to reductionist messages to help them navigate the masses of climate change knowledge. This is problematic, as both graphics and numbers can oversimplify intricacy of science, and thereby become misused or misunderstood. One example is when the IPCC 1.5 °C report was widely misrepresented as saying that there were 12 years left to act to avert the climate catastrophe. In fact, the report stated that in order to stay under 1.5 °C warming, CO_2_ emissions needed to be reduced by half by or before 2030 (i.e. 12 years away from 2018). This led to confusion about when and how to take policy action (Allen 2019; Boykoff and Pearman 2019; Dubash 2020), exacerbating climate anxieties and extreme views, increasing the political polarization on climate change, and generating friction among scientists regarding the most suitable way to frame climate change in communication.

The UNEP Gap Report produced annually since 2010 provides a good illustration of both the power and pitfalls of non-contextual quantitative representation (UNEP 2019*;* Höhne et al. 2020). The articulation of an emissions ‘gap’ has been a politically powerful tool, communicated through one number—the Gigaton gap—and one iconic figure with a range of scenarios. Each report provides examples of promising sectoral actions—energy efficiency, land use, urbanization, and more—to fill this gap. Yet, in each subsequent year, the gap has remained and even grown, leaving unasked, and unanswered, why mitigation potential does not seem to be realized. To do so would require going beyond the discussion of an emissions gap, rooted in science, modelling, and technology, to perhaps examine an ‘implementation gap’, focused on politics, policy studies, sociology, and anthropology. Specifically, it would require understanding, at minimum, the national and local politics of realizing low-carbon transitions, the institutions required to oversee those transitions, and the behavioural changes needed at the level of citizens. But these questions, and their answers, are not amenable to reductionist analysis or acontextual answers.

### (Lack of) epistemological diversity

Since the 1st Assessment Report, published in 1990, the IPCC has come a long way toward diversifying the disciplines in authors, starting with only a handful of scholars outside of the natural sciences, to a more even balance across the Sixth Assessment Report (AR6) with authors, including Coordinating Lead Authors, stemming from both social sciences and humanities to provide greater diversity of perspectives. This is important because Corbera et al. (2016) find that epistemological homogeneity among 5th Assessment Report Working Group III authors contributes to narrowing the range of viewpoints and understandings of solutions to climate change. Similarly, lack of disciplinary representation among IPCC authors contributes to unevenness in the literature and evidence assessed (e.g. Hulme and Mahony 2010). Bjurström and Polk (2011: p. 543), for example, have found that the IPCC, rather than being seen as an interdisciplinary effort, was a ‘loose cooperation between disciplines with limited integration’.

But while bringing theories, methods, and practices from across the range of scholarly disciplines is one step toward interdisciplinarity (Freeth and Caniglia 2020), these efforts can fall flat when the different philosophical underpinnings—i.e. epistemological perspectives—clash and default to ‘disciplinary capture’ by a single perspective (Brister 2016). The problem lies with the way in which different disciplines are not provided equal opportunities to contribute to thinking about solutions in the IPCC. For example, while Working Group II is entitled ‘impacts, adaptation, and vulnerability’, vulnerability is given far less emphasis within the reports, and only became part of the focus in the Third Assessment Report in 2007. Consequently, those researchers working on vulnerability are often devalued by a perspective that emphasizes climate change impacts over the underlying drivers of vulnerability, such as poor development, gender inequality, or racism. Addressing this problem would require an acknowledgement that interdisciplinary approaches need to be a platform for epistemological diversity in order to move away from reductionism, rather than only an exercise in multi-disciplinary representation.

The politically sensitive issue of carbon removal technologies illustrates how the IPCC’s contents are influenced by the dominance of a reductionist perspective. As Vardy et al. (2017) describe, carbon dioxide removal (CDR) and solar radiation management (SRM) techniques are controversial among both scientists and decision-makers due to the considerable known and potentially unknown risks with these measures. Some have observed that by even acknowledging the literature on CDR and SRM, the IPCC was validating a potentially dangerous approach (Gardiner and Fragnière 2018; Frumhoff and Stephens 2018). However, more importantly, the qualitative knowledge on social transformations that could have accompanied the carbon removal narrative in the IPCC scenarios was ‘almost impossible’ to model in a way that would have fit with the scenarios (Vardy et al. 2017: p. 63). Thus, by forcing the knowledge to fit into a reductionist framing, valuable understandings of human behaviour were marginalized. This sends the message that humanity can continue extracting resources and using energy at an ever-accelerating pace without acknowledging that how resources are used will need to change. Even scaling up renewable energy production and use, undoubtedly an important part of the solution to climate change that does not challenge lifestyles and consumption behaviour, can become yet another form of technofix. Knowing that the IPCC has significant influence on scientific knowledge and directions of climate change research in general (Vasileiadou et al. 2011; Goldman et al. 2018), this could also be highly worrying.

In short, the search for solution-oriented, generalizable statements to guide policy lead to an emphasis on forms of knowledge emerging from natural sciences, technology studies, and economics. These understandings do not engage with questions around the social dynamics of climate resilient futures, examined through the social sciences and humanities disciplinary lenses, which engage more with the context-specific politics and the ‘wicked’ nature of the problem, nor with the narratives found in indigenous knowledge. Yet it is precisely contextual understandings that are salient to implementation challenges, particularly in a Paris Agreement world driven by explicitly contextual ‘Nationally Determined Contributions’ to mitigation.

## Revisiting—and rethinking—interdisciplinarity

Interdisciplinarity is the go-to, unquestioned approach to gain more diverse perspectives, because it suggests the opportunity to consider a single problem from a variety of angles, and seek answers through multiple methodologies. However, collaboration is challenged by underlying differences in research methods, and fundamental epistemological disagreements about what constitutes knowledge needed for action. Indeed, whose model of knowledge should come at the forefront of deciding how society needs to respond to climate change? One can argue that ‘such matters are not climate science’ (Bendell 2020), rather that these lie in the domains of political science, sociology, or other human-focused disciplines. But if the nature of the challenge lies well beyond the scope of any single discipline, as climate change does, we have little choice but to grapple with these questions jointly. This is additionally complicated by growing policy pressures to filter statements from a political feasibility perspective. As a result, interdisciplinarity is not insulated from reductionism: quite to the contrary. In an effort to overcome these communications challenges, we often relate results in the form of familiar narratives, eliminating discipline-specific terms that convey the nuances of argument or evidence within the dialect of a single discipline and much meaning is lost.

Importantly, the willingness of all disciplines working on climate change to engage with each other is not a given. Even within the social sciences, competition between how to frame problems and design research means that collaborations can struggle to bear good results (Bracken and Oughton 2006). These clashes have left a room for a positivist worldview to dominate, consequently devaluing qualitative sciences and knowledges rooted in diverse epistemologies. The example of how indigenous knowledge has been sidelined from IPCC reports until recently even though many Indigenous Peoples are at the frontlines of climate change (García-del-Amo et al. 2020; Alexander et al. 2011) demonstrates how ontological disconnects create hierarchies of knowledge, whereby scientific knowledge that can be empirically measured is given greater validity. Encouragingly, there are efforts to link indigenous knowledge with scientific knowledge (Alexander et al. 2011), most notably in the ongoing IPCC AR6. Yet, these processes should not just use indigenous knowledge to ‘validate’ empirical measurements of climate change. To represent such knowledge adequately requires shifting framings away from reductionist science and instead embracing that indigenous knowledge brings other and equally valid understandings of why climate change is happening and what consequences it has.

## New directions for interdisciplinary climate research

We recognize the increasing importance of interdisciplinarity as the climate change crisis worsens (Mooney et al. 2013; Olsen et al. 2013). Research has already identified characteristics of effective interdisciplinary collaborations on climate change. For example, Bruine de Bruin and Morgan (2019) draw on a technique called ‘mental models’ to focus on reorienting collaborations around a common goal and a joint methodology, using improved communication between researchers and their audience, something also underscored by Leigh and Brown (2021) in their study in interdisciplinary projects. Klink et al. (2017) suggest that the key to good interdisciplinary work is continuous evaluation of how researchers interact with each other, the project and the outputs. While retaining our belief in the benefits of interdisciplinary work, and the virtues of encouraging these approaches among new generations of researchers, we also believe we have to do interdisciplinarity better. Here, we propose four interrelated components of a way forward.

First, we have to change the objective of our research from a quest for a unitary vision of the past, present, and future, toward plural and co-existing perspectives. Diverse simultaneous narratives provide greater opportunity for political creativity than single narratives. Indeed, Pearce et al. (2005) suggest that ‘dissensus’ as an entry point, rather than consensus, may allow for greater deliberative decision-making. Studies of the IPCC for example suggest that the search for consensus frequently leads IPCC documents to frame unhelpfully bland conclusions on precisely those issues that are of the greatest policy relevance, and therefore politically charged, losing an opportunity to contribute to learning and debate (Victor 2015; Vardy et al. 2017). This can stem directly from the way that natural and social scientists are forced to make compromises due to their inherently different framings (Vardy et al. 2017). Reflecting the co-existence of the different epistemologies grounding different disciplines allows for productive engagement by a broader cross section. For example, the idea behind large-scale implementation of renewable energy as a climate mitigation strategy needs to be framed in its wider social and environmental impacts. These may include mineral extraction as inputs for manufacturing of renewables with all their exploitative implications of poor communities upstream and land expropriation to make way for solar farms that bring localized problems in the interest of globalized solutions of emission reductions. At the point of renewable energy use, vast areas of land for the expansion of solar farms are likely to present competition over land, often occupied by poor communities, hence giving rise to localized challenges.

Second, we need to draw on more (epistemologically) diverse knowledge sources. An acceptance of contestation and contradiction requires a messier intellectual landscape of climate understanding that emerges from different disciplinary perspectives. This would acknowledge the need for ‘uncomfortable knowledge’ in policy making proposed by Rayner (2012). This means avoiding sanctifying one disciplinary formulation and instead juxtaposing multiple disciplinary formulations. This compels researchers to find new approaches to speak across disciplines, for example, not just drawing on knowledge about human behaviour that can easily be plugged into integrated assessment or other models. Our inherently diverse ways of understanding the world requires us to ask, and answer, questions in different and deeper ways. This epistemological diversity requires us to acknowledge that there are different understandings of *what* knowledge is, and that this influences *how* knowledge is constructed (Gobbo and Russo 2020) and *whose* knowledge counts (England 2015; Haraway 1988). Critically, this also implies a re-working of how researchers communicate with policymakers, the media, and the public. Expectations of definitively stated universalistic statements will need to give way to an appreciation for multiple, contextually embedded messages about future pathways and their implications.

Third, we need to invest more in qualitative research that examines human behaviour. To adequately respond to the call to action, we need to better understand the basis of human responses to climate change. In other words, we need a sound understanding of the implementation gap as much, if not more, than the emissions gap. To do so, we have to go beyond a technical understanding of the potential for mitigation and adaptation, and draw on the social sciences and humanities to explore the factors—political, sociological, and institutional—that help explain whether and how these potentials can actually be realized, as argued by Nightingale et al. (2020). A recent review shows that many adaptation projects are contributing to increased, rather than reduced, vulnerability to climate change due in part to poor understandings of local contexts where projects are being implemented (Eriksen et al. 2021). These findings suggest that adaptation strategies should not be prioritized based on indicators of their implementation feasibility; more important is the consideration of whether they actually reduce the impacts of climate change. Embracing contextual knowledge requires national and local understandings and, as such, works as a defence against reductionism.

Finally, we need to broaden the knowledge base for action-relevant decision-making on climate change—from the current mechanistic approach to setting targets and charting a course alone, to also creating an information base for more regular and informed course correction. Part of the answer lies in encouraging interdisciplinary approaches among new generations of climate change researchers. This also involves asking ‘who speaks for climate change?’ and lessening the focus on reductionist messages and materials. This shift in emphasis comes from a position of humility about future-looking projections, because while our collective behaviour fits certain patterns, it does not fall neatly into deterministic categories. Who could, for instance, have predicted the scale and impact of the Fridays for the Future movement, substantially stimulated by one young pioneer, a feat well-funded NGOs that have been protesting climate change for decades were not able to achieve? Or the lessons about preparedness that we are learning from the COVID-19 pandemic (e.g. Schipper et al. 2020)? In addition to research that allows us to chart a course, we need a climate science that allows us to react flexibly to new developments, and holistically embrace the links between climate change, climate change policy action, and civil society uprisings such as the Black Lives Matter movement (e.g. Sigwalt 2020).

The climate debate requires careful quantitative analysis, without doubt. While there is widespread assumption that numerical values can speak a universal language, reductionist messages can easily be misunderstood and misused if they are stripped of the social and political dimensions within which they are framed—or even worse, if they emerge out of assessment that has marginalized such dimensions. We argue here that current mainstream approaches to interdisciplinarity result in limiting overall contributions from the social sciences and humanities to knowledge on climate change. First, it tends to induce all disciplines to assimilate reductionist sciences, tempted by the incentive that the resultant messages may be more palatable to natural scientists for the purposes of collaboration, consequently eliminating the richness otherwise contributed by these scholars. Second, it may alienate talented qualitative researchers who feel their work is less valued from climate change research, or who cannot find a common entry point for their knowledge. To find adequate solutions to climate change, we need to not only embrace inputs from all disciplines, but also to reframe the questions we ask and the approaches we pursue if we are to inform action without slipping into reductionist framings. To do so, this requires rethinking interdisciplinarity by welcoming a plurality of narratives, embracing multiple disciplinary perspectives, understanding human systems that mediate action better, and actively seeking knowledge that enables adaptive response to surprises.

## Data Availability

Not applicable.
